# Tolerance to nitroglycerin through proteasomal degradation of aldehyde dehydrogenase-2 in a genetic mouse model of ascorbate deficiency

**DOI:** 10.1186/2050-6511-13-S1-A36

**Published:** 2012-09-17

**Authors:** Astrid Schrammel, Gerald Wölkart, Matteo Beretta, Heike Stessel, Kurt Schmidt, Nobuyo Maeda, Bernd Mayer

**Affiliations:** 1 Department of Pharmacology and Toxicology, Institute of Pharmaceutical Sciences, University of Graz, 8010 Graz, Austria; 2 Department of Pathology and Laboratory Medicine, University of North Carolina, Chapel Hill, NC 27599-7525, USA

## Background

L-Gulonolactone oxidase-deficient mice (Gulo(−/−)) were used to study the effects of ascorbate deficiency on aortic relaxation and lowering of blood pressure by nitroglycerin (GTN). Special emphasis was given to changes in expression and activity of vascular aldehyde dehydrogenase-2 (ALDH2), which has been recently characterized as key enzyme of vascular GTN bioactivation.

## Methods

Ascorbate deficiency was induced in Gulo(−/−) mice by ascorbate deprivation for 4 weeks. Some of the animals were concomitantly treated with the proteasome inhibitor bortezomib and effects were compared with ascorbate-supplemented Gulo(−/−), untreated or nitrate-tolerant wild-type mice. Aortic relaxation of the experimental groups to GTN, acetylcholine and the NO donor diethylamine NONOate was studied. Changes in mRNA and protein expression of vascular ALDH2 were quantified by real-time PCR and immunoblotting, respectively, and aortic GTN denitration rates were determined by thin layer chromatography. Ubiquitination of proteins was analyzed by immunoblotting.

## Results

Like GTN treatment, ascorbate deprivation induced vascular tolerance to GTN that was associated with markedly decreased rates of GTN denitration. Ascorbate deficiency did not affect ALDH2 mRNA levels, but reduced ALDH2 protein expression and the total amount of ubiquitinated proteins to about 40% of wild-type controls. These effects were largely prevented by ascorbate supplementation or treating Gulo(−/−) mice with the 26S proteasome inhibitor bortezomib.

## Conclusions

Our data indicate that ascorbate deficiency results in vascular tolerance to GTN via proteasomal degradation of ALDH2. The results support the view that impaired ALDH2-catalyzed metabolism of GTN contributes significantly to the development of vascular nitrate tolerance and reveal a hitherto unrecognized protective effect of ascorbate in the vasculature.

